# 16S rRNA gene sequencing reveals the effect of fluoxetine on gut microbiota in chronic unpredictable stress-induced depressive-like rats

**DOI:** 10.1186/s12991-023-00458-x

**Published:** 2023-08-03

**Authors:** Hui Dong, Xiaowei Tang, Jie Ye, Wenhuan Xiao

**Affiliations:** Teaching hospital of Yangzhou University, Wutaishan Hospital, Yangzhou, Jiangsu Province, China

**Keywords:** Depression, Fluoxetine, Gut microbiota, Illumina sequencing, 16S rRNA

## Abstract

**Objectives:**

Gut microbiota is relevant to the pathogenesis of mental disorders including depression. This study aimed to investigate the influence of fluoxetine (FLX) on the gut microbiota in rats with Chronic Unpredictable Mild Stresses (CUMS)-induced depression.

**Results:**

We confirmed that the 28-day CUMS-induced depression rat model. Chronic FLX administration weakly improved depressive-like behaviors in rats. Illumina 16S rRNA gene sequencing on rat feces showed CUMS increased the relative abundance of *Firmicutes* (60.31% vs. 48.09% in Control, *p* < 0.05) and *Lactobacillus* genus (21.06% vs. 6.82% in control, *p* < 0.05); FLX and CUMS increased *Bacilli* class (20.00% ~ 24.08% vs. 10.31% in control, *p* < 0.05).

**Conclusion:**

Collectively, our study showed that both CUMS and FLX changed the compositions of gut microbiota in rats. FLX and CUMS distinctly regulated the gut microbiota in depressed rats.

**Supplementary Information:**

The online version contains supplementary material available at 10.1186/s12991-023-00458-x.

## Background

Major Depressive Disorder (MDD) is a common but serious mental disorder state characterized by at least one of the symptoms either depressed mood or loss of interest or pleasure most of the day, nearly every day at least 2 weeks [1]. According to WHO (https://www.who.int/news-room/fact-sheets/detail/depression), an estimated 3.8% of the population experience MDD, including 5% of adults (4% among men and 6% among women). It is one of the leading causes of disability worldwide and more than 700,000 people died due to suicide every year [2, 3]. It profoundly affects the individual’s quality of life worldwide.

The microbiota composition of the gut is associated with normal homeostasis, metabolism, cognitive function and disability [4, 5]. The hypothalamic–pituitary–adrenal axis, which has been reported to be hyperactive in patients with depression, is reported to be affected by the microbiota contents of the gut [6–8]. Furthermore, accumulating evidence suggests that the microbial composition of the gut affects the brain functions and psychological state of its host via the “gut-brain axis”, the bidirectional communication between the gut and the brain is mediated by the immune, neuroendocrine, and neuronal pathways [9]. And gut dysbiosis may be associated with mental disorders, such as depression and anxiety [10–13]. Increasing numbers of studies have shown that the ingestion of probiotics (such as *Lactobacillus helveticus* strain NS8 and *L. rhamnosus* JB-1) restores cognitive functioning and improves anxiety/depressive-like behaviors in rats [4, 5]. The participation of gut bacteria in the depressive state could be related to the production of the central nervous system (CNS) inhibitory neurotransmitter, GABA [14, 15]. GABA deficit has been reported to be common in major depressive disorders [15, 16] and chronic stress-induced depression [17]. Meanwhile, Liang et al*.* showed that the administration of probiotics (*L. rhamnosus* and *Bifidobacterium longum*) significantly increases the production of the GABA(A) receptor α5 and δ subunits and improved the cognitive functioning of their study rats [15]. Furthermore, selective serotonin reuptake inhibitor (SSRI) medications, including fluoxetine (FLX), paroxetine and fluvoxamine, have been shown to induce the production and release of GABA in the brain [18–20]. It has also been reported that FLX increases the sensitivity of mammalian cells (mouse fibroblast cell line L929 and HEK-293 T cells) to GABA concentrations by increasing the activity of the GABA(A) receptor α5 subunit [20]. These findings suggest that gut bacteria play a role in the etiology of depression and that they are associated with the observed improvements mediated by SSRIs in depression.

The variable region of bacterial 16S rRNA gene is generally species-specific and can reflect the distance of the genetic relationship between bacteria. High-throughput sequencing of the 16S rRNA gene on the Illumina platform is commonly used to assess microbial diversity in complex samples [21]. Therefore, based on Illumina sequencing of the bacterial 16S rRNA gene, we sought to investigate the effects of long-term FLX treatment on the composition of the gut microbiota of depression rats subjected to CUMS.

## Methods and materials

### Rat depression model and treatments

Forty male Sprague–Dawley (SD) rats (8–10 weeks old, 210–270 g) were obtained from Jiangsu University, laboratory animal center (China). Animals were habituated and then were assigned into four following groups: Control; Control + FLX group, control rats received chronic FLX (1 mg/kg per day, ip.) for 28 days; Depression model group, rats were chronically exposed to interchanging unpredictable mild stressors (cage tilting for 24 h, cold swimming for 5 min (at 4 °C), water or food deprivation for 24 h, tail nip for 1 min (1 cm from the end of the tail), inversion of the light/dark cycle for 24 h, damp sawdust cage for 24 h) for 28 consecutive days, and CUMS + FLX group, rats received both CUMS and FLX for 28 days. Fresh feces samples were collected from individual rats and stored at −80 °C before gut microbiota analysis. All the rats were euthanized (sodium pentobarbital, ip, 80 mg/kg) at the end of behavioral assessment. All the animal experiments were approved by the Animal Care and Ethics Committee of Yangzhou University, China.

### Behavioral assessment

Rat body weight was recorded on days 7, 14, 21, and 28. Behavioral assessment was done with sucrose preference test (SPT) and open-field test (OPT). SPT can reflect anhedonia, a core symptom of depression. SPT were performed using 1% sucrose for 4 h, with a 2-h interval. The sucrose preference was calculated as the sucrose preference (%) = 100% × the amount of sucrose water consumed/the amount of total water consumed. For OPT, rats were placed into a black box (100 cm × 100 cm × 40 cm, 36 rectangles) equipped with a video-tracking system. The 10-min walk distance (m) in the entire system and the center area, the times spent in the centre area (s), and the number of rearings were recorded and analyzed. Walking shorter distance, spending less time in the central area of the black box, and having fewer rearing are considered to be relevant to decreased exploratory activities and anxiety-like behaviors of animals.

### DNA extraction and analysis of gut microbiota

Bacterial DNA was extracted using the QIAamp DNA stool Mini Kit (Qiagen, Germany) from rat feces. DNA samples were diluted (1 ng/μl) and amplified using common PCR primers 515F/806R (the V4 variable region of the 16S rRNA), high-fidelity DNA polymerase (New England Biolabs, USA), and Phusion^®^ High-Fidelity PCR Master Mix kit (New England Biolabs). The PCR products were purified, pooled, and used for the construction of DNA libraries using the TruSeq^®^ DNA PCR-Free Sample Preparation Kit (Illumina, USA). 16 s RNA sequencing was processed on the Illumina MiSeq platform (Illumina, San Diego, USA; 2 × 250 bp PE).

### Data processing

The barcode and PCR primer sequences were removed and the sequencing data were joined using FLASH (v1.2.7; http://ccb.jhu.edu/software/FLASH/). Raw tags were quality-filtered using Qiime (v1.9.1; http://qiime.org/scripts/split_libraries_fastq.html) and chimeric sequences in the clean tags were removed using the UCHIME algorithm (http://www.drive5.com/usearch/manual/uchime_algo.html). Effective tags were clustered using Uparse software (version 7.0.1001; http://drive5.com/uparse/) at 97% identity, and operational taxonomic units (OTUs) were identified. Species annotation of the representative OTU sequences was performed using SILVA’s SSU rRNA database (http://www.arb-silva.de/) by Mothur methods. Sequence taxonomies (70% confidence) in the kingdom, phylum, class, order, family, genus and species were assigned. Multiple sequence alignments were performed using MUSCLE (v3.8.31; http://www.drive5.com/muscle/). The final sequence data for each sample was homogenized, and the alpha diversity estimators, including community richness (Chao1 and ACE indices), community diversity (Shannon and Simpson indices), sequencing depth (Good’s coverage) and phylogenetic diversity (PD_whole_tree) were analyzed. Beta diversity (Unweighted UniFrac distance) was also analyzed based on the homogenized data.

### Statistical analyses

Statistical analyses of experimental data from rats were analyzed using GraphPad Prism 7. All data were expressed as the mean ± standard deviation. Comparisons in the behavior tests were performed using a two-way ANOVA. Differences in sucrose preference and body weight were analyzed using a one-way ANOVA, followed by Tukey’s multiple comparison test. For the sequencing data, alpha diversity between the groups and beta diversity between the samples were analyzed using Qiime software (v1.9.1; http://qiime.org/scripts/split_libraries_fastq.html). The unweighted UniFrac distance was calculated and a UPGMA (unweighted pair group method with arithmetic means) tree was constructed using Qiime software. The principal coordinates analysis (PCoA) results were analyzed using the WGCNA package in R. Differences in alpha and beta diversities were analyzed using the non-parametric Kruskal–Wallis test, and differences in the phylum, class, order, family, genus and species among the different groups were analyzed using MetaStat (permutation *t*-test) with the Benjamini–Hochberg correction controlling the false discovery rate. LDA effect size (LEfSe) analysis was used for biomarker identification. *P* < 0.05 or a corrected *q* < 0.05 was considered statistically significant.

## Results

### FLX and CUMS-induced depressive-like behaviors

**Fig. 1:**
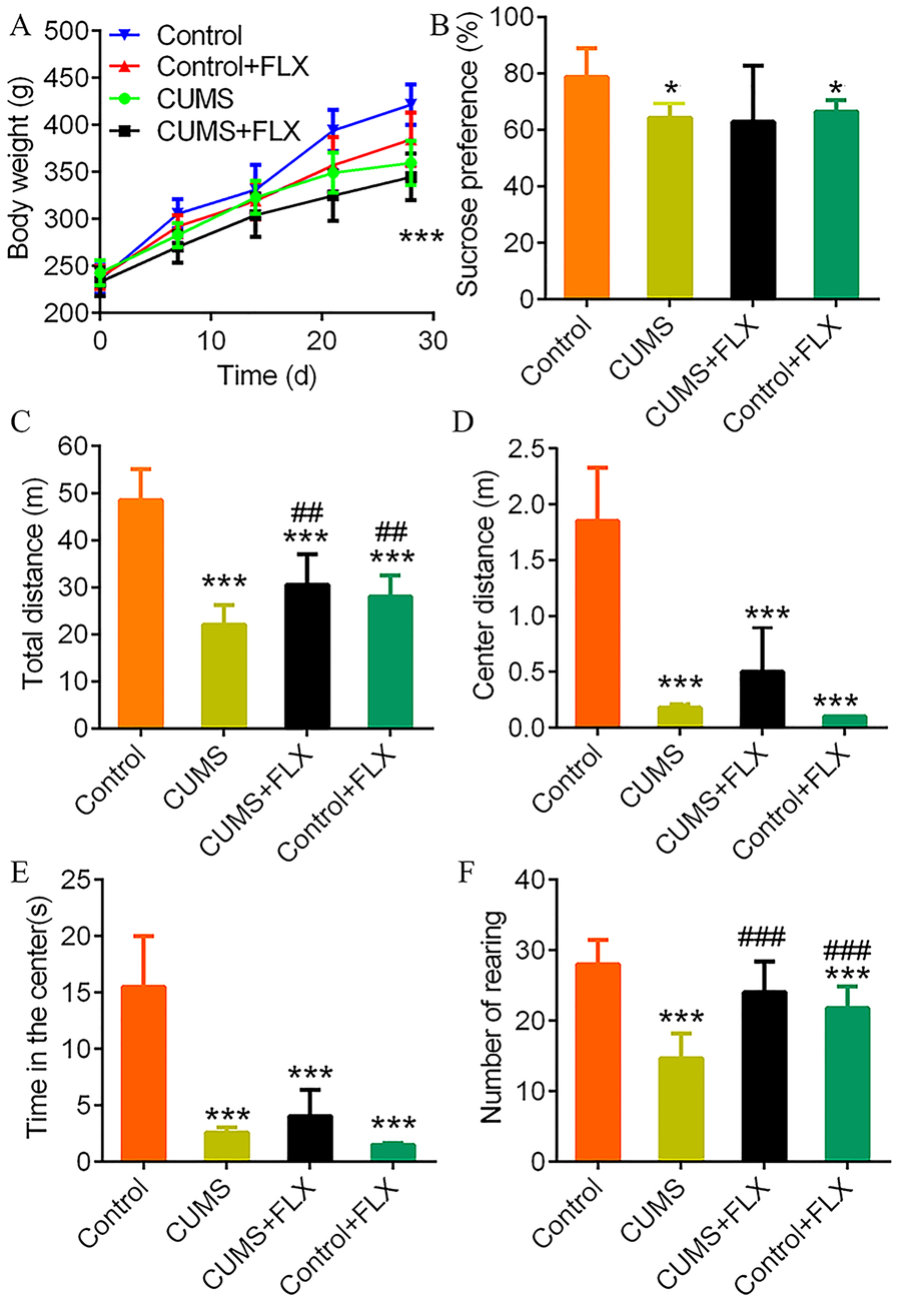
Fluoxetine administration impedes the depressive-like behaviors induced by CUMS. **A**, body weight profiles during the experimental period. **B**, sucrose preference testing on day 14 after the first CUMS. **C**–**F**, results of the open field test on day 28. **: *p* < 0.01 and ***:* p* < 0.001 vs. the Control group by two-way ANOVA. ##: *p* < 0.01 and ###:* p* < 0.001 vs. CUMS group by two-way ANOVA

The body weight of CUMS-treated rats was significantly differences with the controls in the 28 days (Fig. 1A). Sucrose consumption can reflect anhedonia, a core symptom of depression. The CUMS or FLX-treated rats showed significantly decreased sucrose consumption compared to the controls. And FLX administration could hardly prevented this effect in CUMS rats (Fig. 1B). CUMS-induced significant anxiety-like behaviors in rats, walked shorter distances (Fig. 1C and D), spent less time in the center area of the box (Fig. 1E) and had fewer rearing behaviors (Fig. 1F) than rats in the control group (*p* < 0.001, two-way ANOVA, *F* = 669.0). ALL the results confirm that CUMS and FLX both induce depressive/anxiety-like behaviors in normal rats. However, the administration of FLX to rats treated with CUMS could relieve the depressive/anxiety-like behaviors.

### Summary of the Illumina sequencing data

Illumina sequencing captured a total of 2,948,698 highly qualified sequences, with an average length of 253 bp per sequence and an average GC content of 53.49% (Additional file 5: Table S1). All sequences were classified into 5,373 OTUs (1,430 OTUs per sample, Additional file 5: Table S1), including 4,042, 3,407, 2,804, and 2,916 OTUs in the control, CUMS, CUMS + FLX and control + FLX groups, respectively. The 5,373 OTUs were affiliated with 54 phyla, 122 classes, 186 orders, 343 families, 704 genera, and over 2000 species. In total, 1,754 OTUs (32.6%) were identified that overlapped (Additional file 1: Figure S1).

### Diversity analysis of gut microbiota

**Fig. 2:**
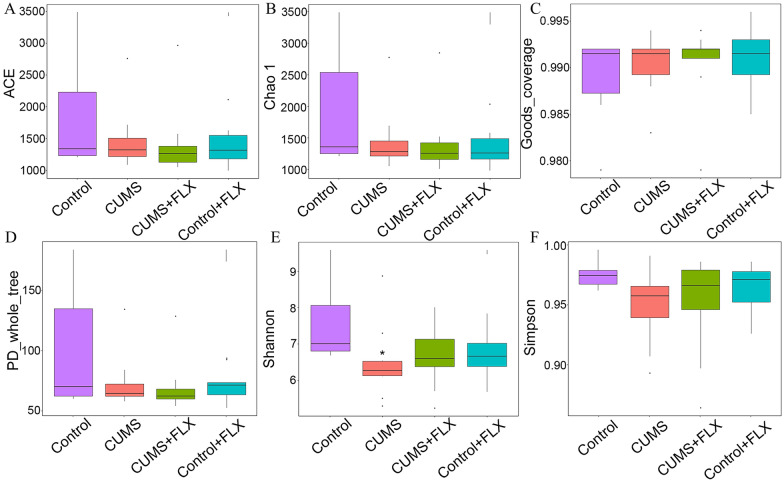
Alpha diversity indices of the OTUs. **A** and **B**, ACE and Chao1 community richness indices. **C**, Good’s sequencing depth coverage estimator. **D**, OUT phylogenetic diversity estimator PD_whole_tree. **E** and **F**, Shannon and Simpson community diversity estimators. *: *p* < 0.05 vs. control by the non-parametric Kruskal–Wallis test

**Fig. 3:**
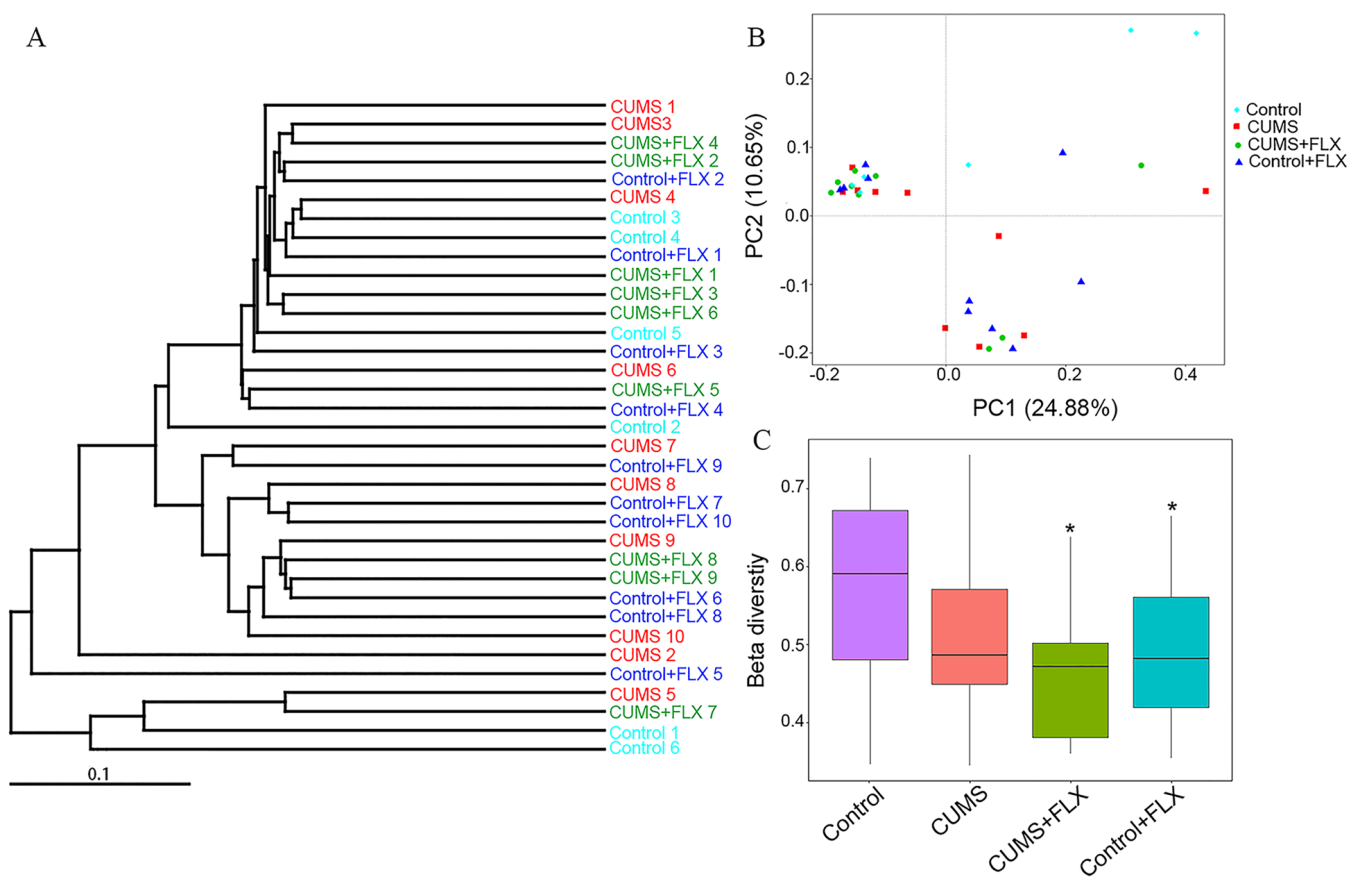
Beta diversity of the OTUs. **A**, UPGMA tree analysis of the relative abundance at the phylum level. **B**, PCoA analysis was used to assess microbial community differences. **C**, beta diversity analysis among groups. *: *p* < 0.05 vs. the Control by the non-parametric Kruskal–Wallis test

There were no differences in the estimators of community richness (Fig. 2A and B), sequencing depth (Fig. 2C) and phylogenetic diversity (Fig. 2D) of the OTUs. CUMS decreased Shannon’s index of community diversity by 12.93% (Fig. 2E) and weakly reduced Simpson’s index (Fig. 2F) compared to the control. The UPGMA trees at the phylum level revealed close relationships and the PCoA analysis showed that the sample data were scattered among 3 quadrants (Fig. 3A and B). FLX administration clearly decreased beta diversity in the rats receiving CUMS compared with the control group (Fig. 3C).

### Significance of CUMS and FLX-altered gut microbiota abundance

**Fig. 4:**
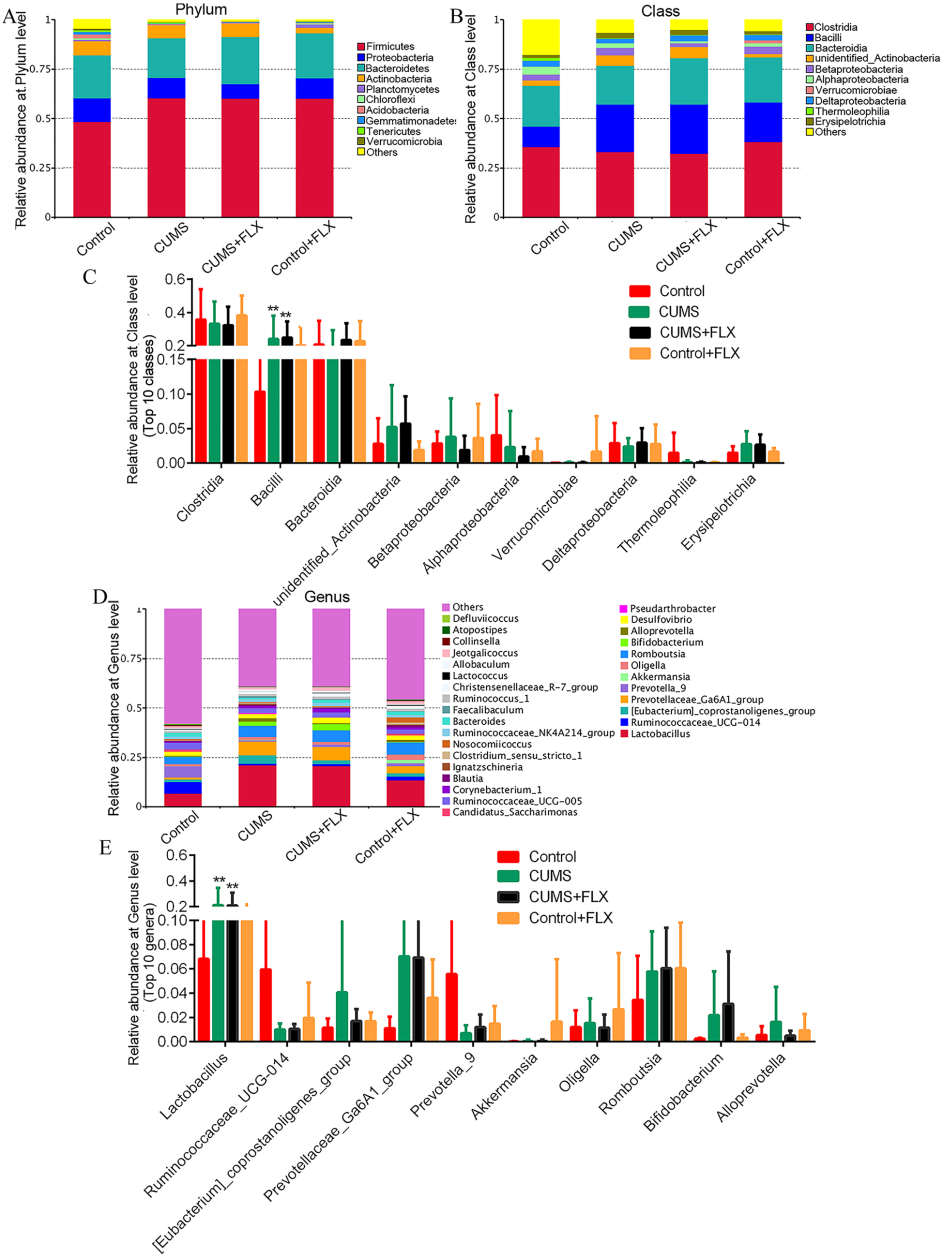
Bacterial community abundance at phylum, class and genus level. **A**, **B**, and **D**, the relative abundances of the OTUs at phylum, class and genus level, respectively. **C** and **E**, the relative abundance of several dominant communities at class and genus level, respectively. *: *q* < 0.05 vs. control. **: *q* < 0.01 vs. the control by MetaStat tests

The main dominant bacterial classes across all samples were *Clostridia* (*Firmicutes* phylum, 32.29–38.16%), *Bacilli* (*Firmicutes* phylum, 10.31–24.95%), *Bacteroidia* (*Bacteroidetes* phylum, 19.64–23.51%) and one unidentified_*Actinobacteria* class (*Actinobacteria* phylum, 1.86–5.71%) (Fig. 4A and B). Comparative analysis showed that CUMS with or without FLX administration increased the abundance of *Bacilli* and unidentified_*Actinobacteria* (Fig. 4C) relative to the control. No significant differences were observed in the relative abundances of the other bacterial classes.

At the genus level, *Lactobacillus* (6.82–21.06%, *Firmicutes* phylum), *Prevotellaceae*_Ga6A1_group (1.11–7.03%, *Bacteroidetes* phylum), *Romboutsia* (3.44–6.04%, *Firmicutes* phylum) and (*Eubacterium*)_coprostanoligenes_group (1.15–4.06%, *Firmicutes* phylum) were the dominant bacteria (Fig. 4D). CUMS with or without FLX administration only resulted in an increased abundance of *Lactobacillus* (21.06% vs 6.82%, *q* < 0.05 by MetaStat test; Fig. 4E). FLX alone did not change the abundances of the dominant bacteria (Additional file 2: Figure S2). Additionally, the MetaStat test revealed statistically significant differences for 11 other genera with small proportions (< 0.1%) in the relative abundances (Additional file 3: Figure S3). This indicates that chronic administration of FLX influenced the diversity of the gut bacterial community in rats.

**Fig. 5:**
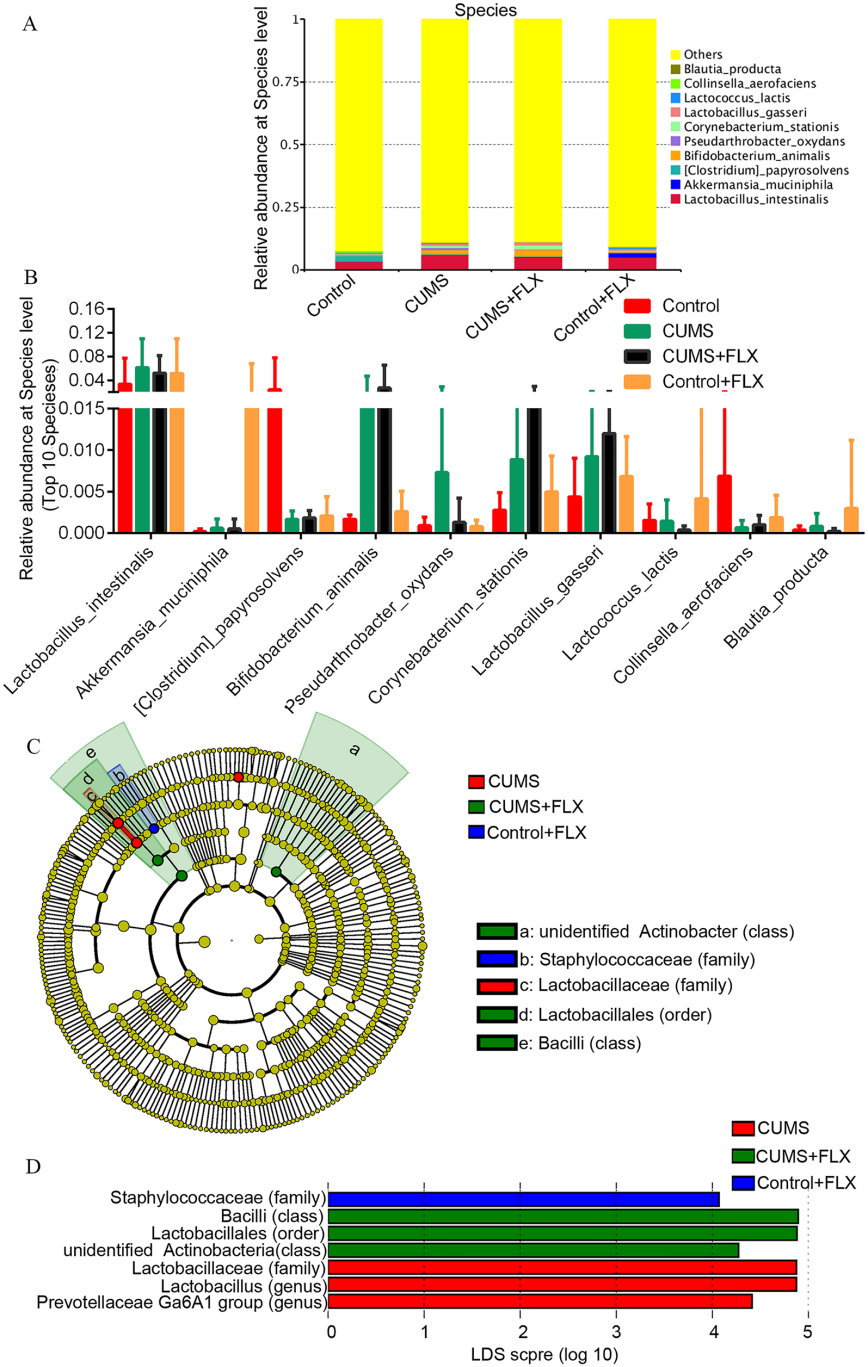
Bacterial community abundance at species level. **A**, the relative abundance of bacterial community at the species level. **B**, relative abundance of the 10 dominant species. **C**, the taxonomic cladogram generated by LEfSe analysis. **D**, LDA scores for taxa enrichment at different level (*n* = 7, threshold > 4)

We also analyzed taxonomic differences in the samples at the species level. *L. intestinalis* (*Firmicutes* phylum, *Bacilli* class) represented the main species in all samples (3.35–6.13%), followed by *B. animalis* (*Actinobacteria* phylum, 0.16–2.71%) and *Akkermansia muciniphila* (*Verrucomicrobia* phylum, 0.02–1.65%) (Fig. 5A and B). The total proportion of species with less than 0.5% abundance was as high as 88.90%-92.65%. MetaStat testing revealed no significant differences in the dominant species among the groups (*p* > 0.05) (Fig. 5D).

### LEfSe analysis for significant gut microbiota

The LEfSe analysis suggested that significant changes in the bacterial community (at class, order, family and genus level) were induced by CUMS and FLX alone or in combination in experimental rats compared to control. The taxonomic cladogram revealed that the unidentified_*Actinobacter* class, a *Bacilli* class and the *Lactobacillales* order (*Bacilli* class) were dominant in the CUMS + FLX group. CUMS alone increased the proportions of the *Lactobacillus* genus (*Bacilli* class) and the *Lactobacillaceae* family (*Bacilli* class). In contrast, FLX alone only increased the proportion of the *Staphylococcaceae* family (*Bacilli* class) in depression-modelled rats (Fig. 5C and Additional file 4: Figure S4). The detailed relative abundance of each significant bacterial community across all samples based on the LEfSe analysis is shown in Additional file 4: Figure S4. These results suggest that CUMS, either alone or in combination with FLX, induced significant differences in the gut microbial communities of the rats.

## Discussion

Traditional antidepressant drugs, such as SSRIs, are effective in 56–60% of the population with depression [22]. Gut microbiota disturbance is associated with impaired cognitive function and anxiety/depressive-like behaviors [4, 5]. Clinical trials and systematic reviews have reported the effective improvement brought about by probiotics in depressive disorders, with the same or higher level of efficiency than SSRIs [23, 24]. The results of the present study have shown that both CUMS and chronic FLX administration led to anxiety-like behaviors and changes in intestinal microbial communities in rats.

We observed that the application of CUMS decreased the community diversity in depresseive rats compared with the controls. The decreased Shannon alpha diversity index for the depression-modelled rats suggested that they had less diverse fecal microbial components than the control rats. We observed that CUMS and FLX in combination or as single treatments altered the composition of the gut microbiota. The proportions of the *Bacilli* class and the *Lactobacillus* genus increased significantly under chronic stress alone or in combination with FLX administration. It was disappointing that chronic FLX administration did not impede these alterations in our rat depression model. It should be emphasized that our results are consistent with studies conducted in adult animals, in which chronic fluoxetine produced anxiogenic-like behavior [25]. They found that chronic fluoxetine administration showed dose-related anxiogenic-like effect in naive rodents, assessed with the elevated plus maze. In contrast, acute administration of SSRIs-like FLX rapidly activated the 5-HT_1B_ receptor, followed by increased AMPA glutamate receptors, and increased potentiation of the excitatory temporoammonic synapse in pyramidal CA1 cells in the hippocampus [26]. These results suggest that the long-term administration of FLX could be not suitable for depression treatment.

Other analyses of microbiological diversity have shown that there are dissimilarities in the intestinal microbial community between patients and rats with depression and normal controls [11, 13, 27]. In a case–control study, bacterial counts for *Bifidobacterium* and *Lactobacillus* in depressed patients were found to be lower than in healthy volunteers [11]. Meng et al*.* showed that levels of the *Lactobacillus* genus decreased in chronic stresses-treated Wistar rats over 28 days [13]. Another study in Wistar rats showed that treatment with 2-week chronic variable physical stressors increased the relative abundance of *Staphylococcus aureus* and *Candida albicans* in these rats [27]. Furthermore, Naseribafrouei et al*.* showed that depression was correlated with a decreased abundance of the *Bacteroidetes* phylum compared to healthy controls [10]. It is possible that these discrepancies might reflect natural differences in the experimental subjects though.

Further analysis using LEfSe revealed that chronic stress with or without FLX in combination increased the level of *Bacilli* bacteria such as the *Lactobacillaceae* family, the *Lactobacillus* genus, and the *Lactobacillales* family. Several probiotic *Lactobacillus* strains like the NS8 *L. helveticus* strain, JB-1 *L. rhamnosus*, *L. helveticus* and HN001 *L. rhamnosus* have been confirmed to improve cognitive function and alleviate stress-induced anxiety/depressive-like behaviors in animal experiments [4, 5, 14, 23, 28, 29]. Indeed, Luo et al*.* showed that ingestion of the probiotic strain of *L. helveticus* NS8 in hyperammonemic rats restored their cognitive functioning and improved their anxiety-like behaviors [4]. Along the same lines, two studies [5, 14] reported that probiotics treatments were able to mediate the improvement of depressive behavior in rat model by elevating the level of GABA and activating GABA signaling in the CNS, indicating that neurobiology and behavior are correlated with the microbiota-gut-brain axis. The increased levels of the *Lactobacillaceae* family and the *Lactobacillus* genus in CUMS-induced rats might indicate that a self-protection mechanism was operating in these animals to compensate for the chronic stress.

The *Lactobacillus* genus with probiotic properties has been found to increase GABA production and expression of the GABA(A) receptor α5 subunit [14, 15]. FLX treatment has also been shown to increase the activity of the GABA(A) receptor alpha5 subunit, neural activity and the sensitivity of mammalian cells to GABA concentrations [20]. The GABA(A) receptor complex is a target of FLX [30], and chronic administration of FLX was found to enhance hippocampal excitatory synaptic transmission in rats through synaptic strengthening, indicating its benefit for the treatment of long-term depression [25]. Our present study revealed that the proportions of four probiotic bacteria, namely *L. gasseri* [31], *L. intestinalis* [32], *L. lactis* [33] and *B. animalis* [34] were not influenced by chronic stress alone or in combination with chronic FLX administration, suggesting that the latter was ineffective in changing the levels of probiotics. It is possible that the levels of these probiotic bacteria were not affected by chronic FLX treatment per se, but by some other mechanism involved in bodily self-defence against chronic stress in rats.

Our study had several limitations. First, chronic FLX treatment only weakly attenuated the CUMS-induced depressive-like behaviors in SD rats compared to control rats, so it is possible that the dose of FLX was insufficient to abolish depressive-like behavior in rats. More reasonable drug administration way should be considered. However, it should be a stress stimuli itself of long-term intraperitoneal injection of FLX for rats. Second, there may be some shortcomings in the experimental design. Namely, a more appropriate design would be to induce depressive-like behavior by chronic stress first and start FLX treatment afterwards. Acute administration of FLX afterward could significantly improve depressive-like behaviors seen in rats. Third, the molecular mechanism underlying the effect of antidepressant treatment on the behavioral changes in rats was not explored, as this was beyond the scope of this study. Four, the open-field test was the only evaluation of depressive-like behavior in rats, without a forced swimming test and the Morris water maze. However, our limited data show that chronic administration of FLX favoured depression and only slightly changed the gut microbial community in model rats.

## Conclusions

Our study has shown that chronic stress and FLX treatment alter the compositions of the gut microbiota in SD rats. Chronic FLX administration did not impede CUMS-induced depressive-like behaviors. The CUMS-related increased levels of the *Lactobacillaceae* family (*Bacilli* class, *Firmicutes* phylum) and the *Lactobacillus* genus (*Bacilli* class) acted as biomarkers for chronic stress-induced depression in SD rats. Thus, we conclude that the composition of the gut microbiota was slightly influenced by FLX administration. Additional studies focused on chronic FLX treatment for depression should be performed to explore the underlying molecular and microbiological mechanisms associated with depression.

### Supplementary Information

**Additional file 1: Figure S1.** Venn image of the OTUs. A total of 1,754 OTUs were shared by the four groups.

**Additional file 2: Figure S2.** Relative abundances of the six dominant communities at the phylum level.

**Additional file 3: Figure S3.** MetaStat *t* test for the difference in relative community abundance at the genus level. RS1, RS2, RS3, and RS4 refer to CUMS, CUMS + FLX, control + FLX, and the control group, respectively. *: q < 0.05 vs. control by MetaStat test.

**Additional file 4: Figure S4.** Detailed relative abundance of the significantly different communities identified in the LEfSe analysis. The solid line represents the mean value, and the dotted line represents the median value.

**Additional file 5: Table S1.** Illumina sequencing data summary.

## Data Availability

The sequencing data generated from the depression rats under different treatments in this study are available in China National GeneBank Databases with the accession number of CNP0000510.
