# Supplementary figures and images for: 16S rRNA gene sequencing reveals the effect of fluoxetine on gut microbiota in chronic unpredictable stress-induced depressive-like rats
Source: Ann Gen Psychiatry. 2023 Aug 3;22:27. doi: 10.1186/s12991-023-00458-x (PMC10398965; doi:10.1186/s12991-023-00458-x)

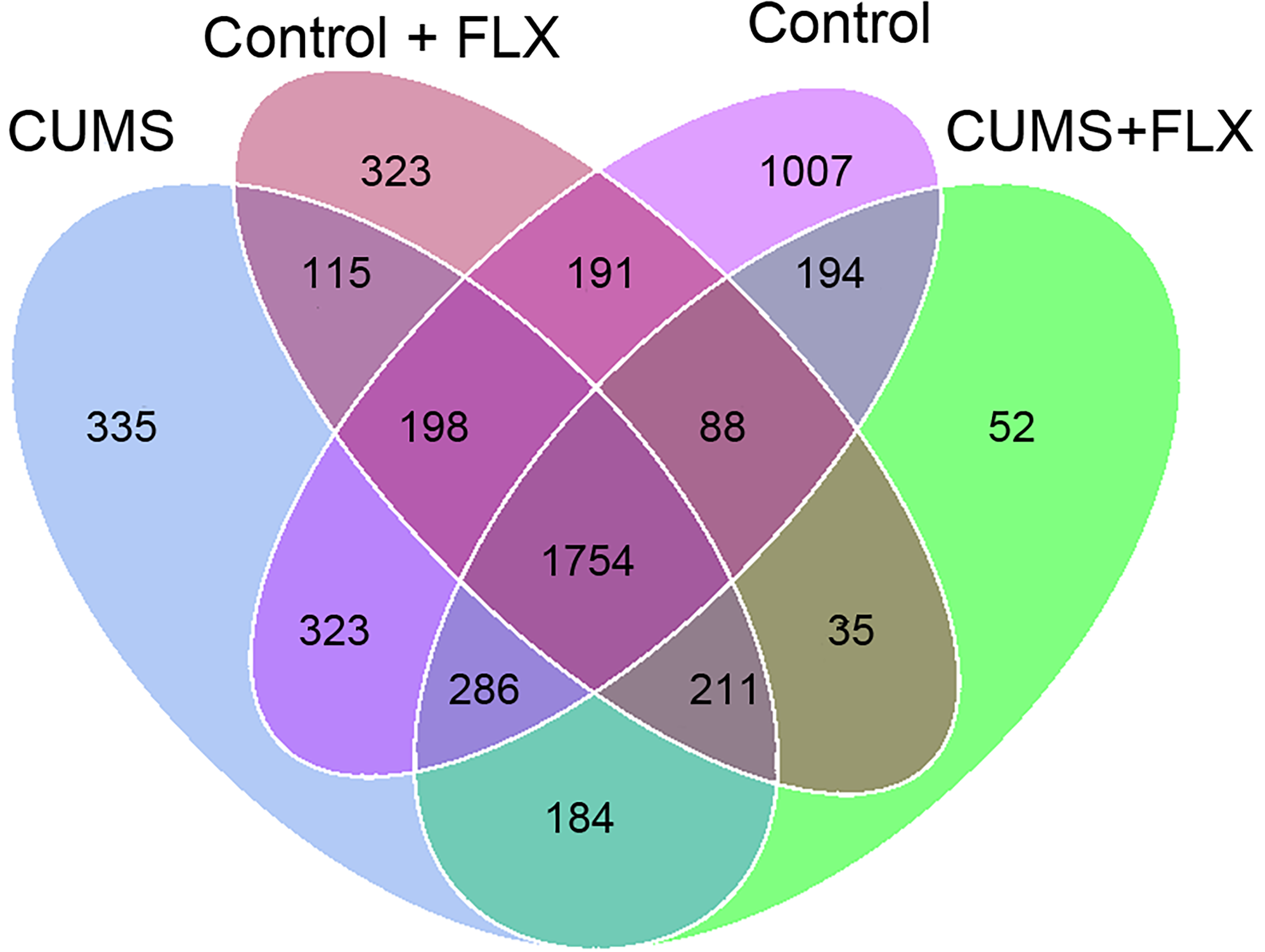

Supplement: Supplementary file 1 — Additional file 1: Figure S1. Venn image of the OTUs. A total of 1,754 OTUs were shared by the four groups. [file 12991_2023_458_MOESM1_ESM.tif]

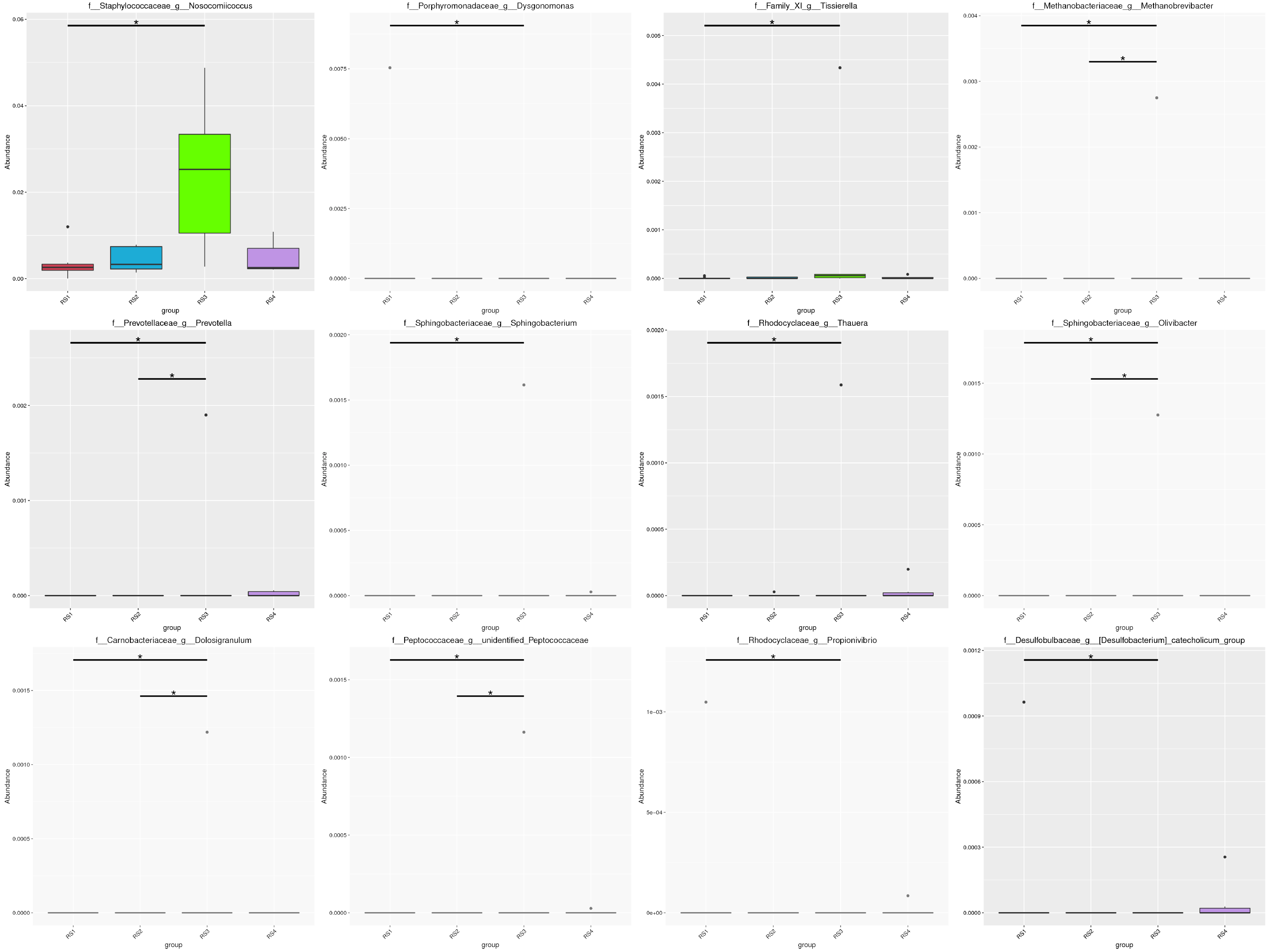

Supplement: Supplementary file 3 — Additional file 3: Figure S3. MetaStat t test for the difference in relative community abundance at the genus level. RS1, RS2, RS3, and RS4 refer to CUMS, CUMS + FLX, control + FLX, and the control group, respectively. *: q < 0.05 vs. control by MetaStat test. [file 12991_2023_458_MOESM3_ESM.tif]

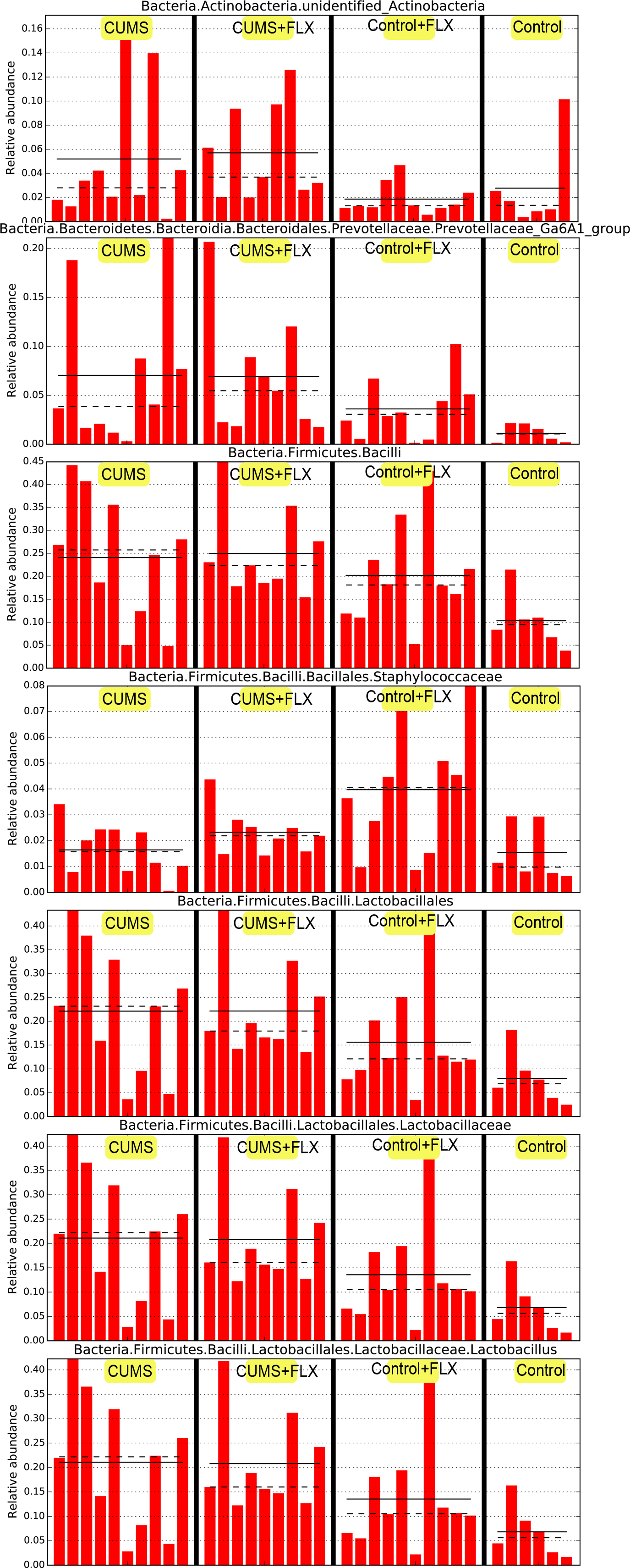

Supplement: Supplementary file 4 — Additional file 4: Figure S4. Detailed relative abundance of the significantly different communities identified in the LEfSe analysis. The solid line represents the mean value, and the dotted line represents the median value. [file 12991_2023_458_MOESM4_ESM.tif]
