# Supplementary material for: 16S rRNA gene sequencing reveals the effect of fluoxetine on gut microbiota in chronic unpredictable stress-induced depressive-like rats
Source: Ann Gen Psychiatry. 2023 Aug 3;22:27. doi: 10.1186/s12991-023-00458-x (PMC10398965; doi:10.1186/s12991-023-00458-x)
Supplement: Supplementary file 5 — Additional file 5: Table S1. Illumina sequencing data summary. [file 12991_2023_458_MOESM5_ESM.docx]

| **Table S1 Summary of the Illumina sequencing data** | | | | |  |  |  |  |  |  |  |  |  |
| --- | --- | --- | --- | --- | --- | --- | --- | --- | --- | --- | --- | --- | --- |
| **Sample ID** | **Raw PE(#)** | **Combined(#)** | **Qualified(#)** | **Nochime(#)** | **Base(nt)** | **Average Length (nt)** | **Q20** | **Q30** | **GC%** | **Effective tags (%)** | **Total tags** | **Taxon tags** | **OTUs** |
| CUMS-1 | 99,375 | 97,847 | 96,585 | 84,324 | 21,289,501 | 252 | 99.24 | 98.4 | 52.75 | 84.85 | 84,342 | 73,909 | 1,237 |
| CUMS-10 | 88,611 | 87,151 | 86,117 | 77,538 | 19,581,813 | 253 | 99.22 | 98.38 | 53.1 | 87.5 | 77,538 | 72,195 | 1,554 |
| CUMS-2 | 84,790 | 83,608 | 82,508 | 81,284 | 20,572,143 | 253 | 99.28 | 98.51 | 52.89 | 95.87 | 81,284 | 77,305 | 1,160 |
| CUMS-3 | 95,093 | 93,724 | 92,641 | 78,466 | 19,809,545 | 252 | 99.21 | 98.35 | 54.04 | 82.52 | 78,466 | 68,346 | 1,099 |
| CUMS-4 | 86,879 | 85,446 | 84,314 | 76,837 | 19,397,525 | 252 | 99.1 | 98.18 | 53.12 | 88.44 | 76,837 | 69,609 | 1,220 |
| CUMS-5 | 89,624 | 86,652 | 85,303 | 72,031 | 18,268,580 | 254 | 99.02 | 98 | 56.16 | 80.37 | 72,031 | 56,378 | 2,574 |
| CUMS-6 | 94,634 | 93,365 | 92,156 | 86,190 | 21,777,668 | 253 | 99.22 | 98.36 | 52.86 | 91.08 | 86,190 | 82,164 | 1,333 |
| CUMS-7 | 80,574 | 78,884 | 77,840 | 65,804 | 16,618,287 | 253 | 99.24 | 98.4 | 53.78 | 81.67 | 65,804 | 53,496 | 1,558 |
| CUMS-8 | 85,850 | 84,567 | 83,549 | 68,061 | 17,187,272 | 253 | 99.17 | 98.29 | 53.25 | 79.28 | 68,061 | 59,852 | 1,292 |
| CUMS-9 | 85,195 | 84,180 | 83,220 | 67,844 | 17,128,108 | 252 | 99.23 | 98.41 | 52.16 | 79.63 | 67,844 | 61,125 | 1,212 |
| CUMS+FLX-1 | 87,999 | 86,503 | 85,506 | 71,141 | 17,962,236 | 252 | 99.22 | 98.37 | 53.42 | 80.84 | 71,141 | 59,909 | 1,178 |
| CUMS+FLX-2 | 87,385 | 86,073 | 84,918 | 74,219 | 18,741,149 | 253 | 99.2 | 98.31 | 53.58 | 84.93 | 74,219 | 62,152 | 1,181 |
| CUMS+FLX-3 | 81,500 | 80,255 | 79,248 | 69,015 | 17,424,487 | 252 | 99.21 | 98.37 | 52.9 | 84.68 | 69,015 | 62,392 | 1,052 |
| CUMS+FLX-4 | 90,414 | 88,824 | 87,710 | 71,476 | 18,049,395 | 253 | 99.25 | 98.44 | 53.84 | 79.05 | 41,476 | 55,705 | 1,255 |
| CUMS+FLX-5 | 98,183 | 96,820 | 95,687 | 85,106 | 21,486,302 | 252 | 99.23 | 98.39 | 53.06 | 86.68 | 85,106 | 74,599 | 1,151 |
| CUMS+FLX-6 | 98,798 | 97,281 | 96,075 | 85,177 | 21,498,521 | 252 | 99.11 | 98.2 | 53.27 | 86.21 | 85,177 | 72,904 | 1,144 |
| CUMS+FLX-7 | 98,051 | 96,046 | 94,823 | 77,866 | 19,669,095 | 253 | 99.18 | 98.29 | 54.37 | 79.41 | 77,866 | 63,159 | 2,539 |
| CUMS+FLX-8 | 91,991 | 90,732 | 89,604 | 73,519 | 18,569,015 | 253 | 99.21 | 98.35 | 52.57 | 79.92 | 73,519 | 64,552 | 1,455 |
| CUMS+FLX-9 | 89,107 | 87,874 | 86,697 | 71,666 | 18,086,197 | 252 | 99.15 | 98.23 | 53.04 | 80.43 | 71,666 | 57,758 | 1,292 |
| Control+FLX-1 | 86,423 | 85,010 | 83,931 | 74,763 | 18,876,410 | 252 | 99.24 | 98.4 | 53.22 | 86.51 | 74,763 | 66,420 | 1,247 |
| Control+FLX-10 | 90,572 | 89,199 | 88,086 | 82,374 | 20,796,203 | 252 | 99.21 | 98.33 | 53.83 | 90.95 | 82,374 | 76,260 | 1,653 |
| Control+FLX-2 | 94,110 | 92,789 | 91,594 | 78,679 | 19,855,810 | 252 | 99.23 | 98.37 | 53.15 | 83.6 | 78,679 | 63,375 | 1,146 |
| Control+FLX-3 | 87,706 | 85,963 | 84,947 | 77,084 | 19,467,495 | 253 | 99.26 | 98.43 | 53.01 | 87.89 | 77,084 | 71,087 | 1,233 |
| Control+FLX-4 | 93,340 | 92,199 | 91,003 | 80,801 | 20,420,379 | 253 | 99.25 | 98.42 | 53.46 | 86.57 | 80,801 | 75,571 | 1,069 |
| Control+FLX-5 | 70,203 | 66,707 | 63,512 | 60,565 | 15,489,526 | 256 | 99.17 | 98.19 | 52.7 | 86.27 | 60,565 | 54,068 | 1,168 |
| Control+FLX-6 | 86,242 | 84,685 | 83,695 | 67,809 | 17,125,733 | 253 | 99.13 | 98.25 | 52.98 | 78.63 | 67,809 | 59,701 | 1,380 |
| Control+FLX-7 | 86,428 | 85,114 | 83,938 | 71,632 | 18,078,185 | 252 | 99.17 | 98.26 | 54.1 | 82.88 | 71,632 | 58,858 | 1,457 |
| Control+FLX-8 | 90,100 | 88,915 | 87,837 | 70,020 | 17,674,123 | 252 | 99.21 | 98.35 | 53.68 | 77.71 | 70,020 | 51,982 | 1,269 |
| Control+FLX-9 | 89,984 | 82,458 | 80,672 | 71,848 | 18,224,787 | 254 | 99.15 | 98.24 | 54.02 | 79.85 | 71,848 | 66,335 | 1,776 |
| Control-1 | 85,965 | 81,455 | 80,046 | 73,310 | 18,525,304 | 253 | 99.14 | 98.22 | 57 | 85.28 | 73,310 | 54,628 | 3,179 |
| Control-2 | 84,949 | 78,681 | 76,800 | 72,837 | 18,565,638 | 255 | 99.06 | 98.1 | 51.94 | 85.74 | 72,837 | 69,026 | 1,265 |
| Control-3 | 84,802 | 83,536 | 82,640 | 78,245 | 19,752,167 | 252 | 99.31 | 98.55 | 53.52 | 92.27 | 78,245 | 73,276 | 1,258 |
| Control-4 | 81,202 | 80,069 | 79,055 | 74,634 | 18,847,580 | 253 | 99.25 | 98.41 | 53.47 | 91.91 | 74,634 | 68,763 | 1,164 |
| Control-5 | 84,274 | 82,818 | 81,728 | 71,175 | 17,975,788 | 253 | 99.19 | 98.3 | 54.12 | 84.46 | 71,175 | 62,440 | 1,142 |
| Control-6 | 60,847 | 57,861 | 44,713 | 40,817 | 11,007,128 | 270 | 98.84 | 97.58 | 53.92 | 67.08 | 40,817 | 35,278 | 2,174 |
| **Total** | 3,071,200 | 3,003,291 | 2,948,698 |  |  |  |  |  |  |  |  |  |  |
| **Average** | 87,748 | 85,808 | 84,248 | 73,833 | 18,679,974 | 253 | 99.19 | 98.3 | 53.49 | 84.03 | 73,833 | 64,388 | 1,430 |
|  |  |  |  |  |  |  |  |  |  |  |  |  |  |
